# Continuous chromosome-scale haplotypes assembled from a single interspecies F1 hybrid of yak and cattle

**DOI:** 10.1093/gigascience/giaa029

**Published:** 2020-04-03

**Authors:** Edward S Rice, Sergey Koren, Arang Rhie, Michael P Heaton, Theodore S Kalbfleisch, Timothy Hardy, Peter H Hackett, Derek M Bickhart, Benjamin D Rosen, Brian Vander Ley, Nicholas W Maurer, Richard E Green, Adam M Phillippy, Jessica L Petersen, Timothy P L Smith

**Affiliations:** 1 Department of Animal Science, University of Nebraska–Lincoln, C203 ANSC, Lincoln, NE 68583, USA; 2 Bond Life Sciences Center, University of Missouri, 1201 Rollins Street, Columbia, MO 65201, USA; 3 Genome Informatics Section, Computational and Statistical Genomics Branch, National Human Genome Research Institute, 9000 Rockville Pike, Bethesda, MD 20892, USA; 4 US Meat Animal Research Center, US Department of Agriculture, State Spur 18D, Clay Center, NE 68933, USA; 5 Gluck Equine Research Center, University of Kentucky, 1400 Nicholasville Rd., Lexington, KY 40546, USA; 6 USYAKS, Livermore, CO 80536, USA; 7 Dairy Forage Research Center, 1925 Linden Drive, ARS USDA, Madison, WI 53706, USA; 8 Animal Genomics and Improvement Laboratory, 10300 Baltimore Ave., ARS USDA, Beltsville, MD 20705, USA; 9 Great Plains Veterinary Educational Center, School of Veterinary Medicine and Biomedical Sciences, University of Nebraska–Lincoln, 820 Road 313, Clay Center, NE 68933, USA; 10 Department of Biomolecular Engineering, University of California, 1156 High St., Santa Cruz, CA 95064, USA

**Keywords:** genome assembly, phasing, *Bos taurus*, *Bos grunniens*, Highland cattle

## Abstract

**Background:**

The development of trio binning as an approach for assembling diploid genomes has enabled the creation of fully haplotype-resolved reference genomes. Unlike other methods of assembly for diploid genomes, this approach is enhanced, rather than hindered, by the heterozygosity of the individual sequenced. To maximize heterozygosity and simultaneously assemble reference genomes for 2 species, we applied trio binning to an interspecies F1 hybrid of yak (*Bos grunniens*) and cattle (*Bos taurus*), 2 species that diverged nearly 5 million years ago. The genomes of both of these species are composed of acrocentric autosomes.

**Results:**

We produced the most continuous haplotype-resolved assemblies for a diploid animal yet reported. Both the maternal (yak) and paternal (cattle) assemblies have the largest 2 chromosomes in single haplotigs, and more than one-third of the autosomes similarly lack gaps. The maximum length haplotig produced was 153 Mb without any scaffolding or gap-filling steps and represents the longest haplotig reported for any species. The assemblies are also more complete and accurate than those reported for most other vertebrates, with 97% of mammalian universal single-copy orthologs present.

**Conclusions:**

The high heterozygosity inherent to interspecies crosses maximizes the effectiveness of the trio binning method. The interspecies trio binning approach we describe is likely to provide the highest-quality assemblies for any pair of species that can interbreed to produce hybrid offspring that develop to sufficient cell numbers for DNA extraction.

## Background

New technologies and algorithms for chromosome-scale genome assembly have improved the contiguity of reference genomes in the past several years [1]. These new methods are more efficient than previous methods, allowing high-quality assemblies of the genomes of a wider variety of organisms, rather than for model organisms only. In addition to increasing assembly efficiency, these technologies have focused on addressing 2 of the foremost challenges of genome assembly: long repetitive regions and heterozygosity of diploid genomes. Repetitive regions are difficult to assemble owing to their low sequence complexity, resulting in gaps in reference genomes [2, 3]. Mitigating this issue, advances in long-read sequencing technologies [4, 5] have facilitated the generation of reads longer than many of these repetitive regions, spanning what otherwise would be assembly gaps [6, 7].

Advances in sequencing technology have thus far not been as successful at resolving heterozygous regions of diploid genomes as they have been at resolving repetitive regions. Heterozygous loci, especially those containing complex structural differences between the haplotypes, add intractable complexity to the assembly graphs used to assemble genomes. Most current long-read genome assemblers, such as canu [8], flye [9], and miniasm [10], choose a random haplotype in each heterozygous region and save the unused haplotype as an alternate, resulting in a single pseudo-haploid assembly containing sequence from both parental haplotypes. Another long-read assembler, FALCON-unzip, uses long reads spanning multiple heterozygous regions to phase the assembly graph as much as possible, but the assemblies it generates still contain numerous haplotype switch errors [11]. The long-range information present in proximity ligation and linked-read libraries has also been used to phase diploid assembly graphs, with mixed results [12, 13].

Trio binning is a new assembly technique that avoids the need for such complex strategies by deconvoluting the problem of diploid genome assembly into a pair of simpler haploid assemblies [14]. Trio binning uses variation present in short reads from 2 parents to sort long reads from their offspring into bins representing either maternal or paternal haplotypes. The long reads in these bins are then assembled independently of one another, resulting in 2 haploid assemblies of higher quality and contiguity than would be possible with a diploid assembly. This method's ability to correctly infer haplotype of origin for long reads from the offspring is dependent on how divergent the 2 parental genomes are because greater divergence results in more places in their offspring's genome where the 2 haplotypes are differentiable. Thus, trio binning produced better results for assembly of an intraspecies hybrid of 2 breeds of cattle (heterozygosity ∼0.9%) than for a human trio (heterozygosity ∼0.1%) [14].

Here, we apply trio binning to an interspecies F1 hybrid of yak (*Bos grunniens*, NCBI:txid30521) and cattle (*Bos taurus*, NCBI:txid9913), 2 species that diverged ∼4.9 million years ago [15] but are capable of producing fertile offspring [16]. The interspecies application of trio binning maximizes the use of heterozygosity to make it easier to bin reads, resulting in high-quality reference genomes for both parental species. The resulting fully phased haploid assemblies of both the cattle and yak genomes contain chromosome arm length haplotigs, representing the most contiguous assemblies to date of large diploid genomes.

## Results

**Figure 1:**
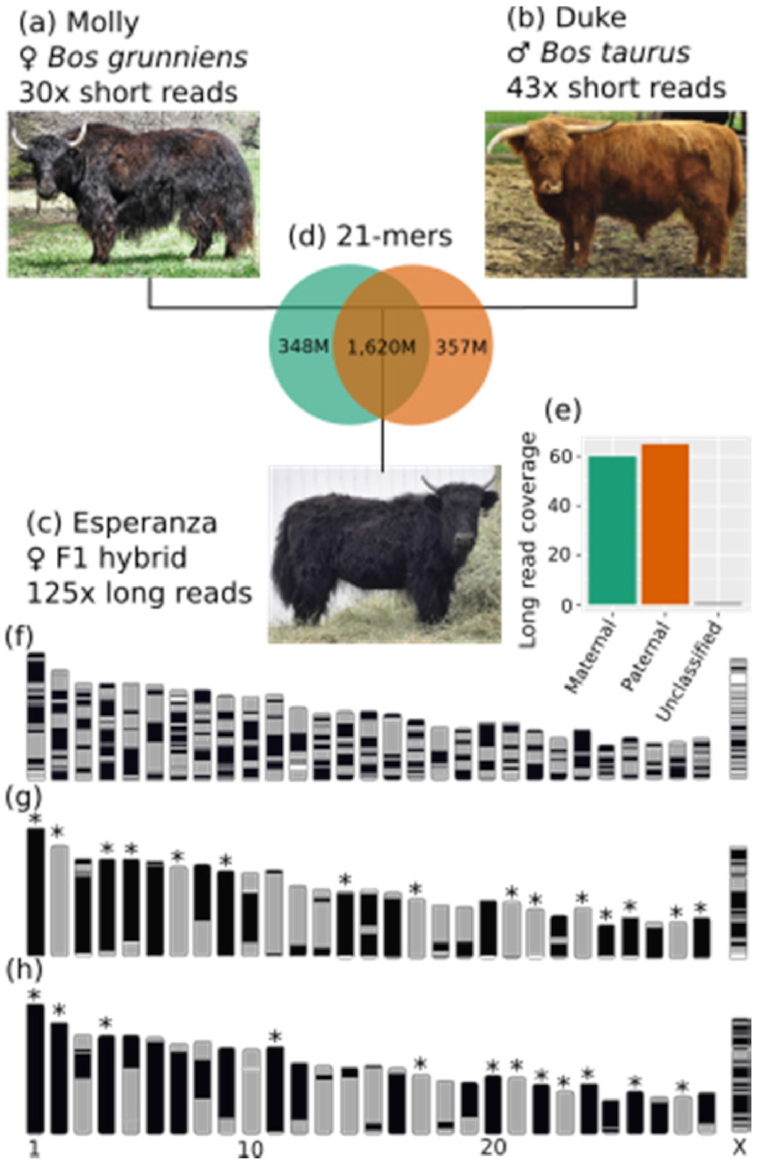
Trio binning of a yak/cattle hybrid. (a–c) We collected short reads from a yak cow and a Highland cattle bull, and long reads from their F1 hybrid offspring. (d) Counts of 21-mers shared by Molly and Duke and those unique to a single parent. (e) Long-read coverage of the maternal and paternal haplotypes after binning reads from Esperanza using 21-mers from (d). (f–g) Ideograms of contigs on chromosomes for (f) ARS-UCD1.2, (g) Esperanza's maternal (yak) haplotype assembly, and (h) Esperanza's paternal (cattle) haplotype assembly, with contigs represented as solid blocks of a single color and full chromosome arms in single contigs noted with an asterisk.

We applied trio binning to a trio consisting of a yak cow (*B. grunniens*) "Molly," a Highland cattle bull (*B. taurus*) "Duke," and their F1 hybrid offspring "Esperanza" (Fig. 1). After verifying Esperanza's parentage (Supplementary Table S1), we sequenced both parents with Illumina short reads and their offspring with Pacific Biosciences long reads. We estimated Esperanza's heterozygosity to be ∼1.2%, compared with ∼0.9% for the cross-breed cattle hybrid assembled by Koren et al. [14], which is consistent with the longer divergence time between yaks and cattle than between indicine and taurine cattle (Supplementary Fig. S1).

Using the short reads from the 2 parents, we found ∼350 million 21-mers unique to each parental line. More than 99% of the total length of the long reads from Esperanza contained 1 or more 21-mers unique to 1 of the parental genomes, allowing them to be sorted into maternal or paternal bins (Fig. 1d and e), each of which were then independently assembled.

The initial contig assemblies of these 2 haplotypes are ultra-continuous (Fig. 1f–h), with contig N50s of 70.9 Mb for the yak haplotype and 71.7 Mb for the cattle haplotype. In addition, more than one-third of the 29 autosomal chromosomes in both assemblies are composed of a single contig: 15 in the maternal and 12 in the paternal assembly. BUSCO [17] analyses of both genomes show most single-copy orthologs present in the initial contig assemblies. A total of 97.1% of single-copy orthologs are present in the maternal assembly, 95.5% of which are both complete and single-copy, while 96.8% of single-copy orthologs are present in the paternal assembly, 95.6% of which are both complete and single-copy.

**Figure 2:**
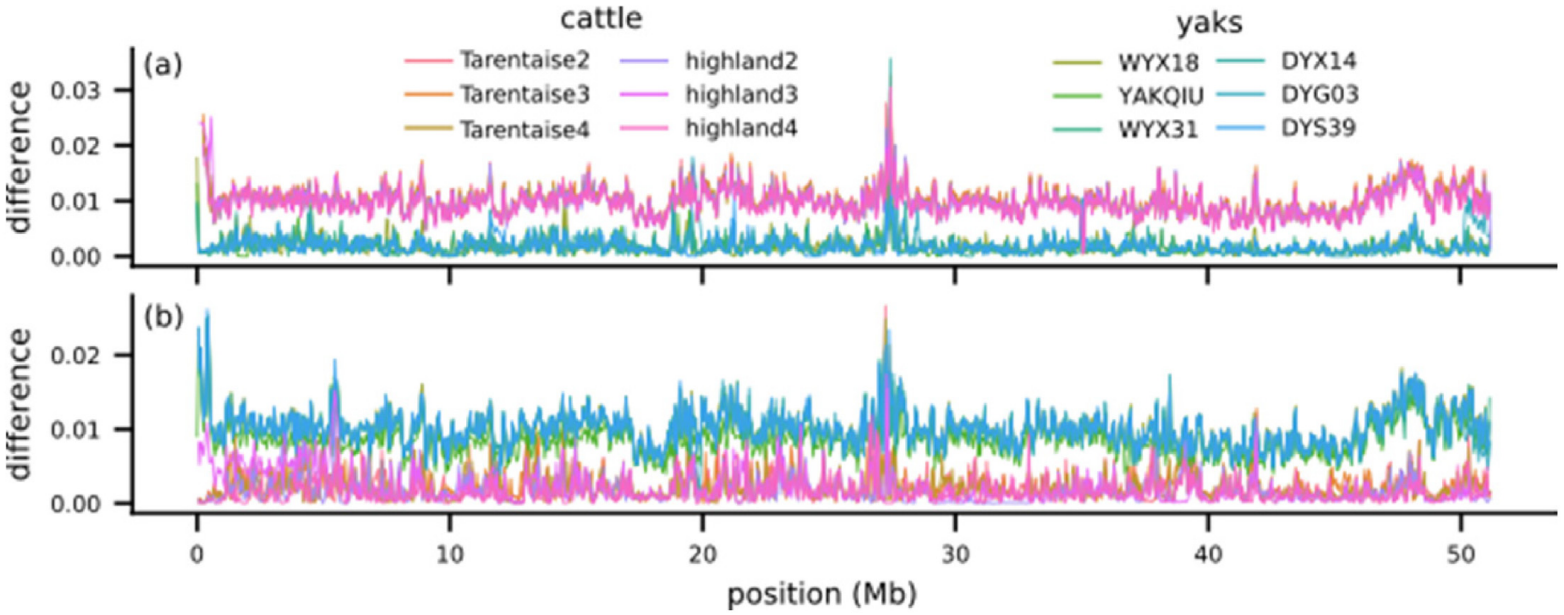
Alignment of 6 cattle and 6 yaks to chr29 of our (a) maternal and (b) paternal assemblies shows that the maternal haplotype assembly is more similar to yak genomes than cattle and the paternal haplotype assembly is more similar to cattle genomes, demonstrating that they are phased correctly.

Trio binning assembly is advantageous not only because removing heterozygous diploidy as a complicating factor leads to more contiguous assemblies but because it results in 2 fully phased assemblies. To confirm that the maternal (yak) assembly and the paternal (cattle) assembly were correctly phased, with no switch errors, we again took advantage of the large divergence between the 2 haplotypes resulting from the interspecies cross by testing the similarity of both assemblies to several cattle and yak individuals (Fig. 2 and Supplementary Figs S2 and S3).

We aligned short reads from 3 Highland cattle, 3 Tarentaise cattle, 2 wild Asian yaks, and 4 domestic Asian yaks to the paternal and maternal assemblies and calculated the number of single-nucleotide polymorphisms (SNPs) for each individual compared to both references in 50-kb windows across the genome. In almost all windows, the mean SNP rate of the 6 cattle is higher than that of the 6 yaks when compared to the maternal yak reference (98.4%), and the mean SNP rate of the 6 yaks is higher than that of the 6 cattle when compared with the paternal cattle reference (99.7%). Notable exceptions to this occur in places such as the beginning of maternal chr11, where all 6 yaks have higher SNP rates compared with the maternal reference than all 6 cattle, indicating that the maternal reference is more cattle-like at these locations. However, the paternal reference is not more similar to the 6 yaks at the same locations, indicating that these are not likely to be haplotype switch errors. Rather, we hypothesize that these are regions of cattle introgression into the maternal genome because introgression among various *Bos* species including cattle and yak is known to be pervasive worldwide [18, 19].

Some chromosomes in both genomes comprise multiple contigs, so scaffolding the assemblies was still necessary. To this end, we sequenced 250 million reads from a Hi-C library created from a tissue sample of Esperanza. The short-read length of a Hi-C short-read library presents fewer chances in each read for finding *k*-mers unique to 1 parent, so we instead aligned all read pairs to both the maternal and paternal haplotype assembly and used alignment scores to bin read pairs. We were able to assign 152 million Hi-C pairs to 1 or the other haplotype using this method, and used the remaining 98 million pairs to scaffold both assemblies. The resulting scaffolds had an N50 of 86.2 Mb for the paternal and 94.7 Mb for the maternal assembly.

Both scaffolded assemblies are highly concordant with the current cattle reference genome (Supplementary Figs S4 and S5). Whole-genome alignment of the 2 assemblies to ARS-UCD1.2 revealed a small number of large (>1 Mb) structural differences between ARS-UCD1.2 and the yak and cattle haplotypes: 4 in the yak and 5 in the cattle haplotype. Further investigation of these discordant segments using a recombination map of cattle [20], an optical map [21], Hi-C heat maps, the location of telomeric repeats, short-read coverage around the breakpoints, and the previous cattle reference UMD3.1 [22] provided sufficient evidence to justify inverting 3 contigs in the maternal assembly and 3 contigs in the paternal assembly.

After assigning scaffolds to chromosomes using the recombination map for autosomes and alignment to ARS-UCD1.2 for the X chromosome, we filled gaps created between contigs during scaffolding and chromosome assignment by aligning binned long reads back to their assemblies using the PBJelly pipeline. This process was able to fill 74 of these gaps in the maternal and 78 in the paternal haplotype assembly, increasing the contig N50s to 79.8 Mb for the maternal and 72.8 Mb for the paternal assembly. We then finalized both assemblies with a polishing step.

Out of 402 identified gaps on the ARS-UCD1.2 reference assembly, our maternal and paternal assemblies conclusively closed 213 and 219 gaps, respectively (Supplementary Table S3). Gap closure was confirmed by the alignment of 500 bp of sequence flanking ARS-UCD1.2 gaps to each assembly and ensuring that the alignments were on the same scaffold, within 100 kb of each other. Gap-flanking sequence could not be placed on the same scaffold (trans-scaffold) in 185 and 179 cases for the maternal and paternal assemblies, respectively, suggesting that ARS-UCD1.2 gaps could be the result of scaffolding errors. Of these trans-scaffold closures, 77 and 110 events in the maternal and paternal assemblies, respectively, were not consistently closed, suggesting structural differences between the assemblies that may indicate true differences between species or individuals.

The intersection of repetitive element annotations with gap-flanking sequence revealed that most ARS-UCD1.2 gap regions may have been caused by discrepancies in scaffolding of contigs that were terminated by L1 long interspersed nuclear elements (LINEs) (Supplementary Table S4). These events were followed closely by BovB repetitive elements, which may have also terminated a large proportion of contig ends. While the association of repetitive elements in gap-flanking sequence points towards a potential cause for the gap region in ARS-UCD1.2, we cannot rule out the possibility that transposition of L1 LINEs, BovB, and other active retroelements may have been spuriously detected in this analysis. Inconsistency of the closure status of gaps in the sire and dam assemblies (Supplementary Table S5) suggests that some of these regions may have been sites of non-allelic homologous recombination that had occurred after the divergence between *B. taurus* and *B. grunniens*.

**Figure 3:**
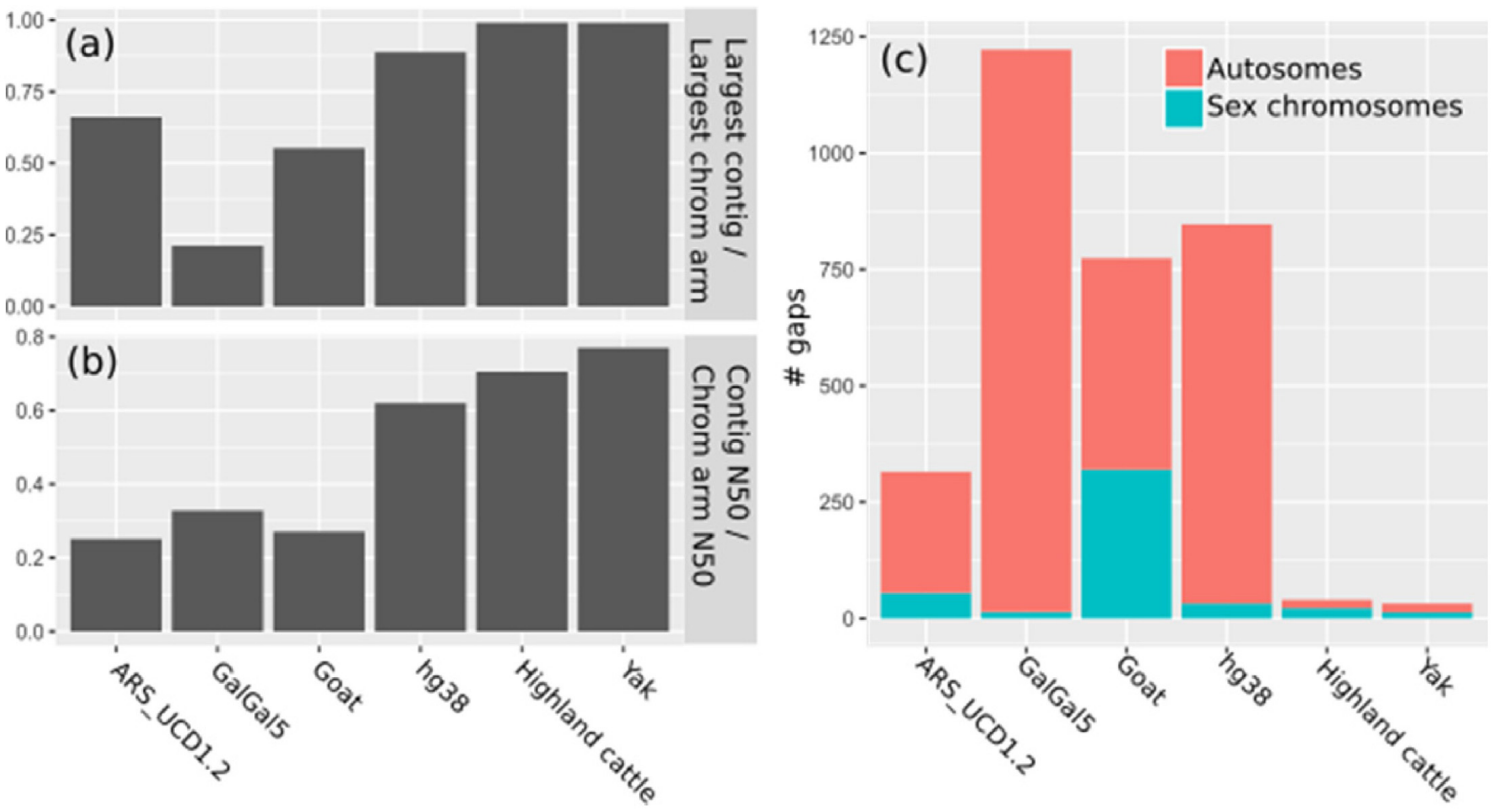
Comparison of trio Highland cattle and yak assemblies to current cattle, chicken, goat, and human reference assemblies, based on ratio of largest contig size to largest chromosome arm size (a), ratio of contig N50 to chromosome arm N50 (b), and number of gaps in autosomes and the major sex chromosome, i.e., X in cattle, yak, goat, and human and Z in chicken (c). We note that the number of gaps in hg38 is somewhat inflated owing to its gapped assembly of centromeres.

The final assemblies of both the cattle and yak genome contain the largest contigs and the fewest gaps of any current assembly of a large diploid genome (Fig. 3). Both cover the largest 2 chromosome arms, the q-arms of chr1 (158 Mb) and chr2 (136 Mb), with a single contig. The maternal yak assembly has 19 gaps on autosomes and 13 gaps on the X chromosome; the paternal Highland cattle assembly has 18 gaps on autosomes and 22 gaps on the X chromosome. For comparison, the current cattle reference ARS-UCD1.2 has 260 gaps on autosomes and 55 on the X chromosome; both assemblies reduce this number of gaps by nearly a factor of 10. Furthermore, our trio assemblies of yak and cattle are comparable or superior to other vertebrate reference genomes in terms of contig N50, number of gaps, and size of largest contig compared with size of largest chromosome arm.

Trio binning also resolves heterogeneous loci into haploid sequences. BOLA, the bovine major histocompatibility complex, is a set of highly diverse loci on chr23 containing variants associated with infectious disease susceptibility [23, 24]. The trio assembly of the cattle contains all 4 subclasses of BOLA in a single contig (Supplementary Fig. S6).

## Discussion

The application of trio binning to a yak/cattle hybrid trio demonstrates that this method is capable of producing highly accurate reference assemblies more continuous than those currently available for species with large diploid genomes. The initial contig N50s of the maternal yak and paternal cattle assemblies, at 70.9 and 71.7 Mb, respectively, are larger than the contig N50s of the current references for yak (20.4 kb) [15] and cattle (ARS-UCD1.2, 25.9 Mb) [25]. Our assemblies are also more continuous than the previous trio binning assemblies of bovines, at 23.3 and 26.6 Mb for the maternal and paternal haplotypes of an Angus × Brahman cross [14]. Thus, our initial haplotig assemblies, even before scaffolding and gap-filling, represent large improvements over existing assemblies of the cattle and yak genomes.

These assemblies not only represent large improvements compared with the current cattle reference genome but are more contiguous by some measures than even the highest-quality reference genomes of organisms such as human (hg38), chicken (galgal5), and goat [26]. For example, the largest contig in hg38 is a 132-Mb contig containing most of the 140-Mb q-arm of chr4, whereas more than one-third of the q-arms of the all-acrocentric autosomes in our assemblies comprise a single contig.

Moreover, these assemblies used as input only long reads from a single individual and short reads from its parents, including the mitochondrial genomes, which were assembled from parental short reads. We also used a Hi-C library to scaffold the assemblies and various orthogonal data types to correct errors in the scaffolding and assign scaffolds to chromosomes, but many chromosomes in both haplotypes were assembled into single contigs in the initial long-read assembly and thus did not require these additional data types. By comparison, recent chromosome-scale assemblies of other non-model mammals such as horse [27] and goat [26] required many additional data types, such as Sanger sequence, bacterial artificial chromosome clones, Chicago libraries, optical maps, and linked reads, to achieve their levels of contiguity and composition. We used a pre-existing genetic map for validation of our assemblies, but the high contiguity and accuracy of our scaffolded but otherwise unedited contig assemblies demonstrates that long reads plus a Hi-C library are sufficient for producing high-quality assemblies using trio binning.

Furthermore, our results demonstrate that it is now technologically feasible to assemble full chromosome arms gap-free with only long reads. The remaining gaps in our assembly are likely the result of repetitive regions such as ribosomal DNA, centromeres, and large segmental duplications too large to be spanned by the long reads we have, but the ever-increasing maximum read lengths achievable with SMRT [4] and nanopore [5] sequencing continue to surpass the sizes of new repetitive regions. We predict that these improvements to existing technologies, along with algorithmic advances such as those that enabled assembly of the human Y centromere [28], will therefore make gap-free assemblies of vertebrate genomes possible in the near future.

These assemblies are not only highly contiguous but have the additional advantage of being fully haploid rather than pseudo-haploid as in most current reference assemblies of large diploid genomes. This is especially valuable in highly heterogeneous regions of the genome where the 2 haplotypes in an individual are most likely to be divergent. We show that the haploid assemblies produced by trio binning can fully resolve difficult-to-assemble heterogeneous loci such as MHC without the need for additional phasing data. This technique is likely to represent a large benefit in the assembly of out-bred or wild vertebrate species that are known to produce viable hybrids.

Trio binning using a cross-species hybrid, in addition to allowing for easier binning of long reads through increased heterozygosity, also has the advantage of producing reference genomes for 2 species with long reads from only a single individual. Thus, this approach will be especially useful for comparative genomics studies in which contiguous haploid reference genomes for 2 related species can be used to identify evolutionary breakpoints with high accuracy.

## Conclusions

Our assembly of chromosome-length haplotigs for the yak and cattle genomes using trio binning suggests that trio binning is the best approach currently available for assembling the genomes of diploid organisms that either can be cross-bred with a closely related species or at least have enough population structure within the species to allow breeding 2 unrelated parents with divergent genomes. The application of the trio binning method to an interspecies cross represents a significant advance over existing methods because the high heterozygosity present in an interspecies cross results in phased diploid assemblies of higher continuity than currently possible with any other method and because it allows the creation of reference genomes for 2 species from a single individual. While many organisms of biological interest are polyploid or unable to be bred in a controlled setting, many model organisms and other highly studied species would be good candidates for trio binning. We therefore expect this method soon to be used to assemble new reference genomes for a variety of species.

## Methods

### Sample collection and preparation

All animal protocols were approved by the Institutional Animal Care and Use Committee of the University of Nebraska–Lincoln, an AAALAC International Accredited institution (IACUC Project ID 1648). Whole blood (EDTA) was collected via jugular venipuncture from the Highland bull and yak cow. Tissue sampling of the yaklander heifer was conducted after euthanization using pentobarbital administered intravenously (1 mL/4.5 kg). Lung tissue was flash frozen and stored at −80°C until DNA isolation and sequencing.

### Long-read library preparation and sequencing

Genomic DNA was extracted from Esperanza's lung tissue using the high salt extraction method as described previously [26]. The DNA was converted into sequencing libraries using the SMRTbell Express Template Prep Kit (Pacific Biosciences, Menlo Park, CA) as directed, except without any shearing step. Three libraries were prepared, 1 with a 25-kb cut-off setting on the BluePippin instrument (Sage Science, Beverly, MA) and 2 with a 30-kb cut-off setting. The libraries were sequenced with 44 cells on a Sequel instrument (Pacific Biosciences Sequel System, RRID:SCR_017989) using Sequel Sequencing kit v2.1 chemistry (Pacific Biosciences, Menlo Park, CA).

### Short-read library preparation and sequencing

Genomic DNA from Esperanza's lung tisse (used also for long-read sequencing, above) was converted into sequencing libraries using the TruSeq DNA PCR-Free LT Library Preparation Kit (Illumina Inc., San Diego, CA) as directed. The shearing was conducted on a Covaris S220 instrument (Covaris Inc., Woburn, MA) with setting to 350-bp fragment size. The same procedure was used to create libraries for parental and unrelated yak samples, except the DNA was prepared from blood using a standard phenol: chloroform extraction as described previously [29]. Sequencing was performed by 2 × 150 bp paired-end sequencing on a NextSeq500 instrument (Illumina NextSeq 500, RRID:SCR_014983) using High Output Kit v2 (300 cycles) kits.

### Hi-C library preparation and sequencing

A sample consisting of 33.36 mg frozen lung tissue from Esperanza was removed from cold storage and homogenized by chopping with a sterile scalpel. The resulting lung paste was transferred to a microcentrifuge tube along with 1 mL phosphate-buffered saline. Paraformaldehyde (EMS Cat. No. 15,714) was added to a final concentration of 3%, and the sample was vortexed briefly before rotation for 20 minutes at room temperature. Collagenase from a Dovetail Hi-C Library Preparation Kit (Catalog No. 21,004) was added to the cross-linked tissue and incubated for 1 hour at 37°C in an agitating thermal mixer. The liquid phase was taken from this reaction and brought to a final concentration of 1% sodium dodecyl sulfate (SDS).

Cross-linked chromatin was bound to SPRI beads and washed thoroughly before digesting with DpnII (20 U, NEB Catalog No. R0543S) for 1 hour at 37°C in an agitating thermal mixer. Biotin-11-dCTP (ChemCyte Catalog No. CC-6002–1) was incorporated by DNA Polymerase I, Klenow Fragment (10 U, NEB Catalog No. M0210L) for 30 minutes at 25°C. Following another wash, intra-aggregate ligation with T4 DNA Ligase (4,000 U, NEB Catalog No. 0202T) was carried out overnight at 16°C. Cross-links were reversed in an 8% SDS solution with Proteinase K (30 g, Qiagen Catalog No. 19,133) for 15 minutes at 55°C followed by 45 minutes at 68°C. After SPRI bead purification, DNA was split into 2 replicates and sonicated to an average length of 350 bp using a Diagenode Bioruptor NGS platform.

Sheared DNA samples were run through the NEBNext Ultra II DNA Library Prep Kit for Illumina (Catalog No. E7645S) to perform the end preparation, adaptor ligation with custom Y-adaptors, and SPRI bead purification steps before biotin enrichment via Dynabeads MyOne Streptavidin C1 beads (ThermoFisher Catalog No. 65,002). Indexing PCR was performed on streptavidin beads using KAPA HiFi HotStart ReadyMix (Catalog No. KK2602) and subsequently size selected with SPRI beads.

We sequenced 250 million reads of this library on a 2 × 151 bp run of an Illumina NextSeq500 (Illumina NextSeq 500, RRID:SCR_014983) using High Output Kit v2 (300 cycle) kits (Illumina Inc., San Diego, CA).

### Heterozygosity estimation

The heterozygosity of Esperanza was estimated using Genomescope (GenomeScope, RRID:SCR_017014) [30].

### Parentage confirmation

Using the Illumina whole-genome shotgun sequence data generated for this project, as well as other data already published to the public domain, we calculated the number of sites relative to the bovine reference genome that did not follow the expected pattern of inheritance. For example if the sire was homozygous for allele A, the dam homozygous for allele B, and the progeny homozygous for B, in the absence of a genotyping error, this pattern suggests that the reported sire is not in fact the sire. We expect some genotyping errors [31, 32], but whatever exclusions are identified when analyzing the verifiable trio should be dwarfed in number when 1 of the actual parents is swapped in the analysis with an unrelated animal. For this comparison we did trio analysis of the yak × cattle offspring versus the reported Highland sire and yak dam as well as the reported dam versus 4 unrelated Highland bulls, and the reported sire versus an unrelated yak dam.

The UnifiedGenotyper (GATK, RRID:SCR_001876) [33] was used in gt_mode = DISCOVERY to analyze the mapped datasets (bam files) for cattle × yak progeny in turn versus a prospective sire/dam pair to identify sites polymorphic in the trio, then genotype those positions producing a vcf file. A custom java program was written to search the dataset for exclusions. Given the nature of this cross it was expected that the majority of the sites identified would be those specific to the interspecies mating. Specifically, we ignored all polymorphic sites with the interspecies cross signature of the bovine sire homozygous for an allele A (consistent with the bovine reference allele), the yak dam homozygous for allele B (likely consistent with the allele fixed in yak), and the progeny heterozygous A/B. Because this pattern would be common to any cattle × yak mating, it would not be suitably specific for a parentage test. The sequence data from the animals not part of the trio were generated for another study with a much lower fold coverage requirement. The coverage for these other animals is on average ∼14×. It has been demonstrated previously (cattle and sheep genotyping articles) that a genotyping accuracy of ∼98% can be attained at this level of coverage. The ∼2% error rate in that work was attributable to an undersampling of the second allele for heterozygous genotypes or allele dropout in the assay-based genotyping platform. This will have the effect of increasing the rate of exclusions in those animals not reported to be the parents, but it should be at a rate of ∼1% of the total genotypes analyzed. This error amounts to a small contribution to the observed exclusion count for the negative controls. The results are presented in Supplementary Table S1. The reported yak dam produced ∼12-fold fewer exclusions than the negative control dam (4.99% vs 0.42%) and the reported Highland sire produced ∼32-fold fewer exclusions when compared with the negative control sires (6.24%, 5.70%, 5.76%, and 7.06% vs 0.19%). These results indicate a correct parental assignment.

### Contig assembly

The trioBinning scripts [34] were used to classify the reads. Briefly, meryl from canu 1.7.1 was used to count all parental*k*-mers. The *k*-mers specific to both the maternal and paternal haplotype were identified via the meryl difference command. Finally, any paternal *k*-mer occurring ≥6 times and any maternal *k*-mer occurring ≥4 times were retained for classification:

meryl -B -C -m 31 -s maternalIlluma.fa -o mom -threads 28 -memory 60000
meryl -B -C -m 31 -s paternallIlluma.fa -o dad -threads 28 -memory 60000
meryl -M difference -s dad -s mom -o dad.only
meryl -M difference -s mom -s dad -o mom.only
meryl -Dt -n 6 -s dad.only |awk “{if (match($1, “>”)) {COUNT = substr($1, 2, length($1)); } else {print $1" "COUNT}}” |awk “{if ($NF < 100) print $0}” > dad.counts
meryl -Dt -n 4 -s mom.only |awk “{if (match($1, “>”)) {COUNT = substr($1, 2, length($1)); } else {print $1" "COUNT}}” |awk “{if ($NF < 100) print $0}” > mom.counts

Reads with no parental marker were not used in downstream analysis. Classified reads were assembled with Canu 1.7.1 with the patch for truncated consensus (git commit e42d54d4f1b1133b8e944b09733806bfe63bc600) command “genomesize = 2.8g” “correctedErrorRate = 0.105” “cnsErrorRate = 0.15” “corMhapSensitivity = normal” “ovlMerThreshold = 500.”

### Scaffolding

We preprocessed the Hi-C reads by trimming to the DpnII junction sequence GATCGATC. To separate the junction-split Hi-C read pairs into maternal and paternal bins, we aligned all reads to both maternal and paternal contig assemblies using bwa mem v0.7 (BWA, RRID:SCR_010910) [35] with default parameters. We then ran the classify_by_alignment program v0.2.1 [36] to determine on the basis of the “AS” tag of the resulting bam files whether each read pair aligned better to the maternal contigs, the paternal contigs, or both equally. If the read pair aligned better to one haplotype than the other, we used it to scaffold only this haplotype, but if it aligned equally well to both, we used it to scaffold both haplotypes. We then ran SALSA2 v2.2 [37] to scaffold both assemblies using the parameters “-e GATC -m yes.”

### Quality control

To find possible mis-assemblies in the scaffolds, we aligned them to ARS-UCD1.2 [25] using mashmap v2.0 [38] with parameter “–perc_identity 95.” We also aligned probes from a recombination map of cattle [20] to the scaffolds using bwa mem v0.7 [35]. We examined resulting alignments for each chromosome for evidence of disagreements between our assembly and ARS-UCD1.2 or the recombination map. Where such disagreements existed, we used the combination of evidence from Hi-C heat maps, ARS-UCD1.2, the recombination map [20], an optical map [21], telomeric repeat location, the previous cattle reference UMD3.1 [22], and short-read coverage around the breakpoint to determine whether there was sufficient evidence to edit our assembly to better match the reference. In total, we inverted the orientation of 3 haplotigs in the paternal assembly and 3 haplotigs in the maternal assembly.

### Chromosome assignment

We used the alignments of recombination map probe sequences as described above to order and orient scaffolds onto chromosomes. Because the recombination map does not include the X chromosome, we used the mashmap alignments between our assemblies and ARS-UCD1.2 to order and orient scaffolds onto the X chromosome.

### Gap filling

We filled remaining gaps in each assembly using the PBJelly pipeline [39], which we modified for compatibility with current versions of the software upon which it depends: BLASR [40] v5.3.2 and networkx [41] v2.2. This modified pipeline is available at GitHub [42].

### Gap analysis

Gap-flanking sequence consisting of 500 bp of sequence from the 5′ and 3′ ends of each gap region was extracted from the ARS-UCD1.2 reference genome. These flanking sequences were aligned to the sire and dam haplotig assemblies using bwa mem v0.7 [35] and checked for consistency. If both gap-flanking sequences were on the same scaffold, were within 100 kb [43] distance of each other, and had no intersecting gaps from the same assembly, the gap was considered closed. Repetitive elements were identified using RepeatMasker (RepeatMasker, RRID:SCR_012954), with the settings “-q,” “-species cow” and “-no_is.” Repeat annotations were converted to bed coordinates and were intersected with gap-flanking regions using Bedtools [43].

### Polishing

Arrow from SMRTanalysis v5.1.0.26412 (pbcommand v0.6.7, arrow 2.2.2 ConsensusCore v1.0.2, ConsensusCore2 v3.0.0, pbalign version: 0.3.1) was used via the ArrowGrid pipeline [44]. Only classified reads were used to polish each haplotype. Initial contigs were polished with 2 rounds of Arrow. Final gap-filled assemblies were again polished with 2 rounds of Arrow, using SMRTanalysis v6.0.0.47841 (pbcommand v1.1.1, arrow 2.2.2, ConsensusCore v1.0.7, ConsensusCore2 v3.0.0, pbalign version: 0.3.1).

### Telomere location

We tested for the presence of telomeric repeats at the ends of each chromosome sequence by counting the number of exact occurrences of the telomeric repeat sequence (TTAGGG) in the final kilobase of each chromosome. We consider a chromosome sequence to end with a telomere if the telomeric repeat sequence occurs ≥5 times in the last kilobase, which is roughly equivalent to *P* < 10^−7^. We used the script count_telo_repeats [45].

### Phasing confirmation

We confirmed that our assemblies are phased correctly by comparing both references to several yak and cattle genomes. We downloaded short reads from SRA for 3 Highland cattle, 3 Tarentaise cattle, 4 domestic yaks, and 2 wild yaks. Supplementary Table S2 lists the IDs and SRA accessions of these individuals. We aligned short reads to both maternal and paternal haplotype assemblies using bwa mem v0.7 [35] with default parameters and sorted alignments and removed PCR duplicates using samtools sort and rmdup [46] with default parameters. Finally, we called SNPs and calculated window SNP rates, which we define as (No. of homozygous SNPs + 0.5 * No. of heterozygous SNPs)/(No. of bases genotyped in window), using samtools mpileup output piped to a custom script pileup2windows [45]. We used the mpileup parameters “-Q 20 -q 20” to exclude low-quality base calls or alignments from the pileup, and we did not call SNPs for positions where the sequencing depth was <2.5th percentile or >97.5th percentile position-depth for that sample.

## Availability of Supporting Data and Materials

The datasets generated and/or analyzed during the present study are available in the NCBI BioProject repository under accessions PRJNA551500 and PRJNA552915. All supporting data and materials are available in the *GigaScience* GigaDB database [47].

## Additional Files

**Supplementary Figure S1**. Histogram of 21-mer coverage in short reads from Esperanza gives a genome heterozygosity estimate of ∼1.2%.

**Supplementary Figure S2**. Comparison of 12 yak and cattle genomes to maternal haplotype assembly.

**Supplementary Figure S3**. Comparison of 12 yak and cattle genomes to paternal haplotype assembly.

**Supplementary Figure S4**. Dot plots of alignment of maternal/yak assembly vs ARS-UCD1.2, the current cattle reference genome, by chromosome. Blue and red colors denote forward and reverse matches, respectively.

**Supplementary Figure S5**. Dot plots of alignment of paternal/highland cattle assembly vs ARS-UCD1.2, the current cattle reference genome, by chromosome. Blue and red colors denote forward and reverse matches, respectively.

**Supplementary Figure S6**. Contiguous haplotypes of chromosome 23 in ARS-UCD1.2 vs the trio assembly of Highland cattle, alongside the locations of the 4 subclasses of BOLA, the bovine MHC. The 4 subclasses are all on the same haplotig of the Highland cattle assembly, while they are on different contigs in ARS-UCD1.2.

**Supplementary Table S1:** Results of verification of Esperanza's parentage.

**Supplementary Table S2**: SRA accessions of yak and cattle libraries used for phasing confirmation.

**Supplementary Table S3:** Intersection of repetitive elements with gaps.

**Supplementary Table S4:** Frequency of repetitive elements flanking gap regions. Values are the number of repetitive elements identified in gap-flanking regions or the intervening sequence between flanking sequences.

**Supplementary Table S5:** Consistency of gap closures between sire and dam assemblies.

giaa029_GIGA-D-19-00342_Original_Submission

giaa029_GIGA-D-19-00342_Revision_1

giaa029_GIGA-D-19-00342_Revision_2

giaa029_Response_to_Reviewer_Comments_Original_Submission

giaa029_Response_to_Reviewer_Comments_Revision_1

giaa029_Reviewer_1_Report_Original_Submission Qiye Li -- 11/15/2019 Reviewed

giaa029_Reviewer_2_Report_Original_Submission Zhonglin Tang, Ph.D. -- 11/17/2019 Reviewed

giaa029_Reviewer_2_Report_Revision_1 Zhonglin Tang, Ph.D. -- 1/28/2020 Reviewed

giaa029_Supplemental_Tables_and_Figures

## Abbreviations

BLASR: Basic Local Alignment with Successive Refinement; bp: base pairs; BUSCO: Benchmarking Universal Single-Copy Orthologs; BWA: Burrows-Wheeler Aligner; EDTA: ethylenediaminetetraacetic acid; GATK: Genome Analysis Toolkit; kb: kilobases; LINE: long interspersed nuclear element; Mb: megabases; MHC: major histocompatibility complex; NCBI: National Center for Biotechnology Information; NGS: next-generation sequencing; SDS: sodium dodecyl sulfate; SMRT: single molecule real time; SNP: single-nucleotide polymorphism; SPRI: solid phase reversible immobilization; SRA: Sequence Read Archive.

## Ethics Approval and Consent to Participate

All animal procedures were approved by the Institutional Animal Care and Use Committee of the University of Nebraska—Lincoln (UNL, Protocol 1648). UNL is an AAALAC International accredited institution.

## Competing Interests

The authors declare that they have no competing interests.

## Funding

This work was supported by ARS Project No. 3040-32000-034-00D. E.S.R. and sample collection costs were supported by funding from an Enhanced Research Collaboration grant from the University of Nebraska–Lincoln, Institute of Agriculture and Natural Resources, Agricultural Research Division and the USDA Meat Animal Research Center. D.M.B. was supported by USDA CRIS project No. 5090-31000-026-00-D. S.K., A.R., and A.M.P. were supported by the Intramural Research Program of the National Human Genome Research Institute, National Institutes of Health. A.R. was also supported by the Korean Visiting Scientist Training Award (KVSTA) through the Korea Health Industry Development Institute (KHIDI), funded by the Ministry of Health AND Welfare (HI17C2098).

## Authors’ Contributions

This study was conceived and designed by T.P.L.S. with input from J.L.P. and A.M.P. T.H. and P.H. identified the animals and collected ante mortem samples; B.V.L., M.P.H., and J.L.P. were responsible for handling the euthanasia and tissue collection post-mortem. T.P.L.S. was responsible for long-and short-read genomic and RNA sequencing, except N.W.M. and E.S.R. prepared Hi-C sequencing libraries. E.S.R., A.R., and S.K. generated the assemblies. E.S.R., S.K., D.M.B., and B.D.R. conducted quality control and assessment of the assemblies. T.S.K. performed additional computational analyses. M.P.H., B.V.L., and R.E.G. provided additional input. E.S.R., S.K., A.M.P., J.L.P., and T.P.L.S. wrote the manuscript. All authors read and approved the final manuscript.
