# Supplementary figures and images for: Continuous chromosome-scale haplotypes assembled from a single interspecies F1 hybrid of yak and cattle
Source: Gigascience. 2020 Apr 3;9(4):giaa029. doi: 10.1093/gigascience/giaa029 (PMC7118895; doi:10.1093/gigascience/giaa029)

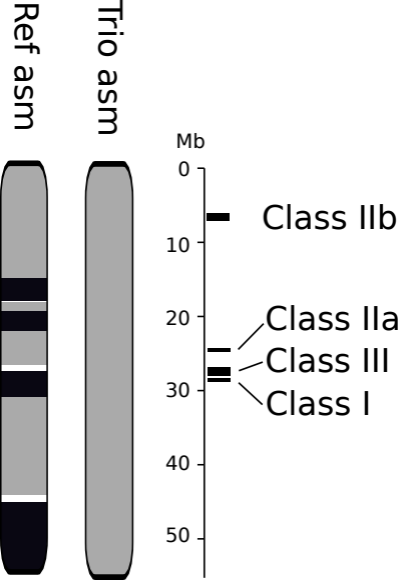

Supplement: giaa029_Supplemental_Tables_and_Figures [file giaa029_supplemental_tables_and_figures.zip › bola.pdf]

# Esperanza

len:2,963,603,168bp uniq:62.2% het:1.19% kcov:9.21 err:0.35% dup:0.457% k:21

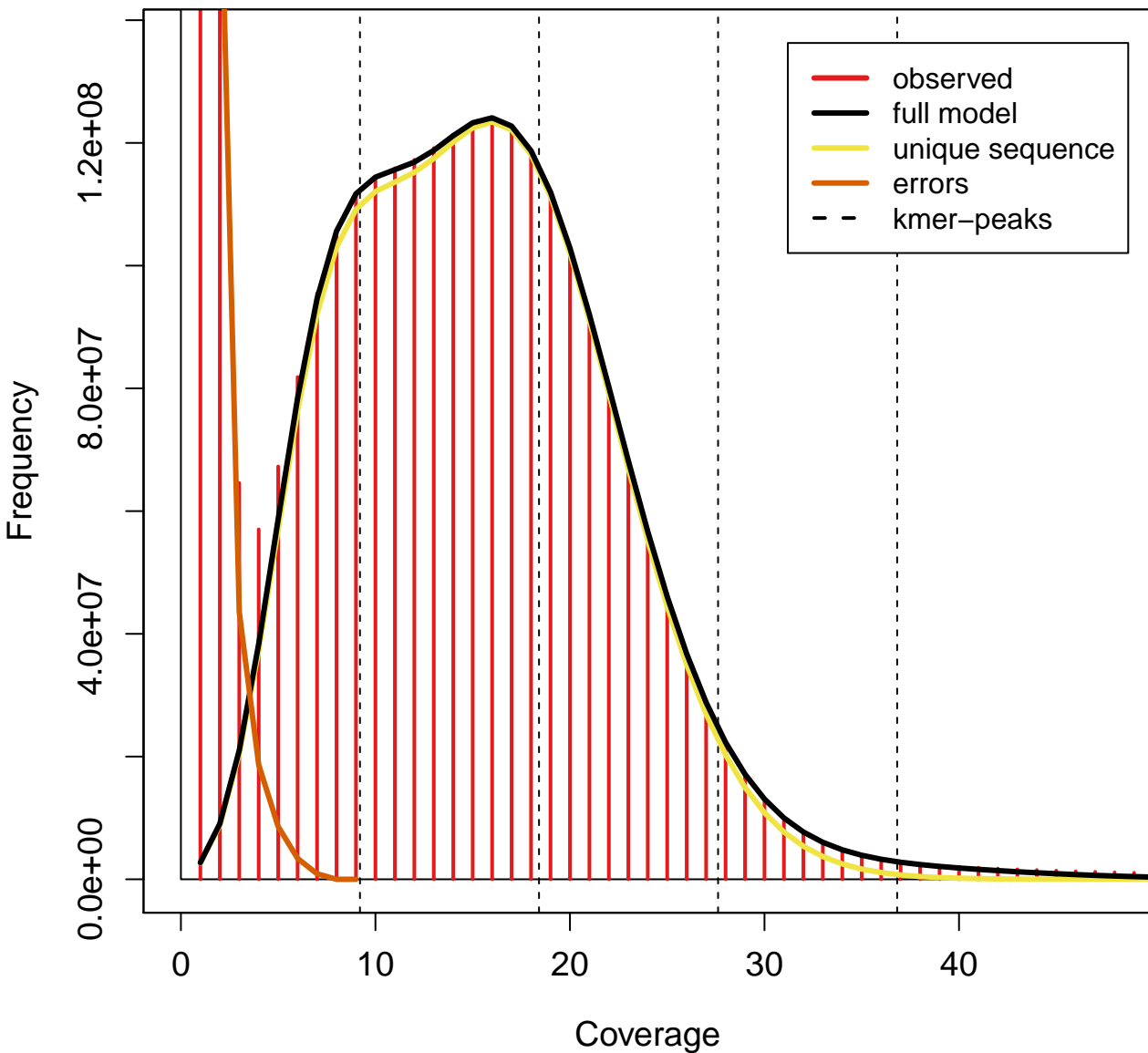

Supplement: giaa029_Supplemental_Tables_and_Figures [file giaa029_supplemental_tables_and_figures.zip › Supp_Fig_S1.pdf]

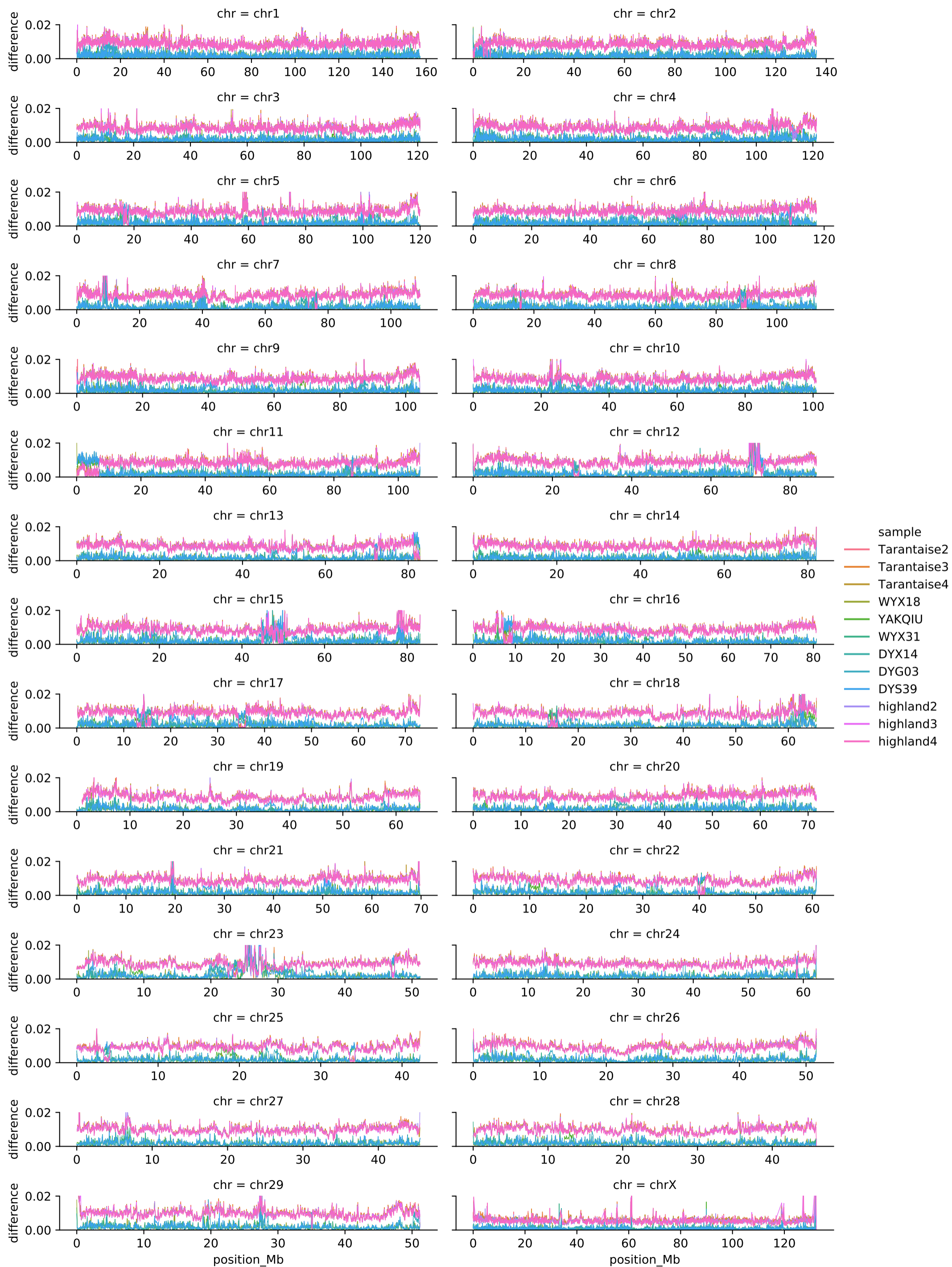

Supplement: giaa029_Supplemental_Tables_and_Figures [file giaa029_supplemental_tables_and_figures.zip › Supp_Fig_S2.pdf]

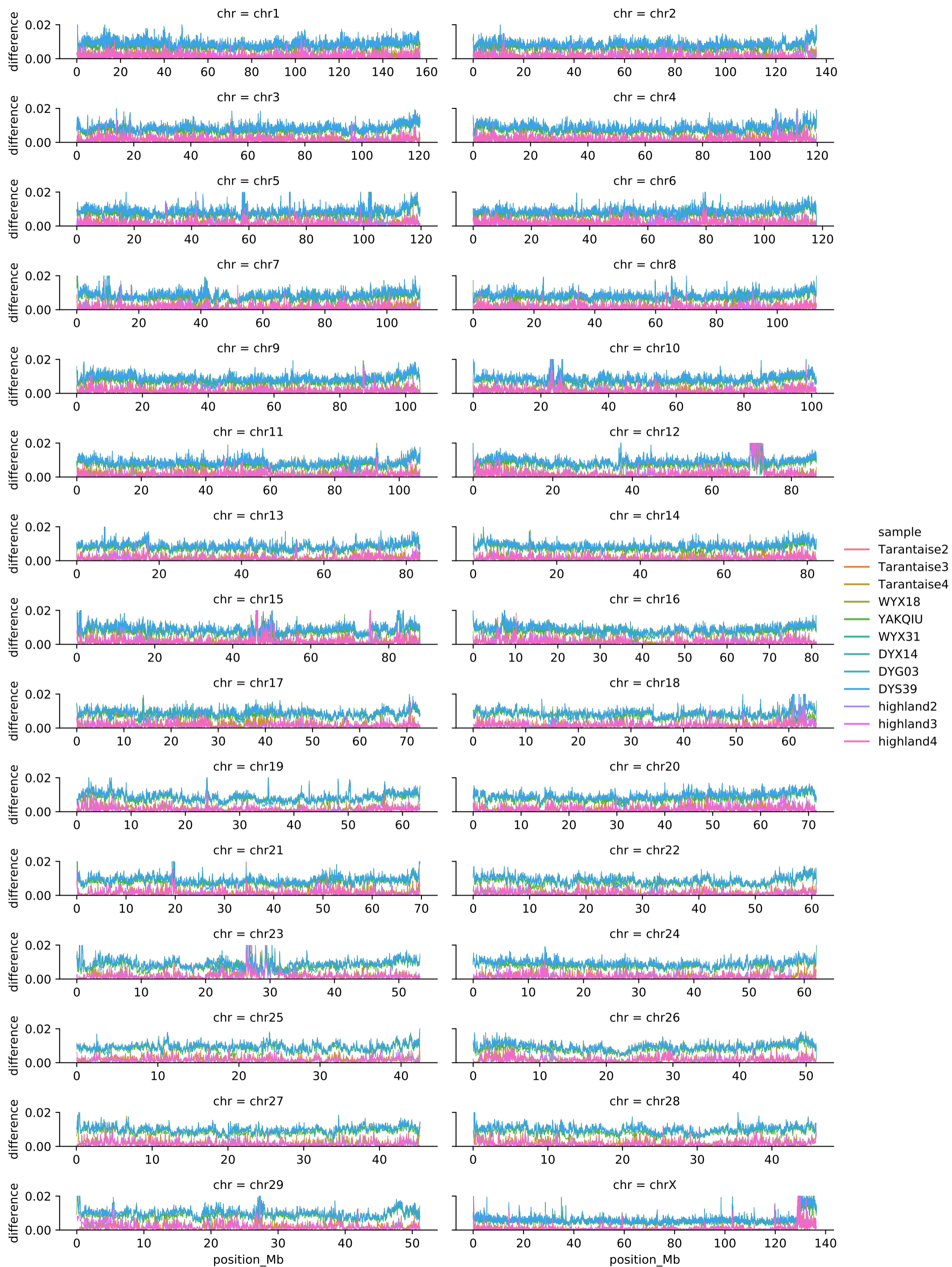

Supplement: giaa029_Supplemental_Tables_and_Figures [file giaa029_supplemental_tables_and_figures.zip › Supp_Fig_S3.pdf]

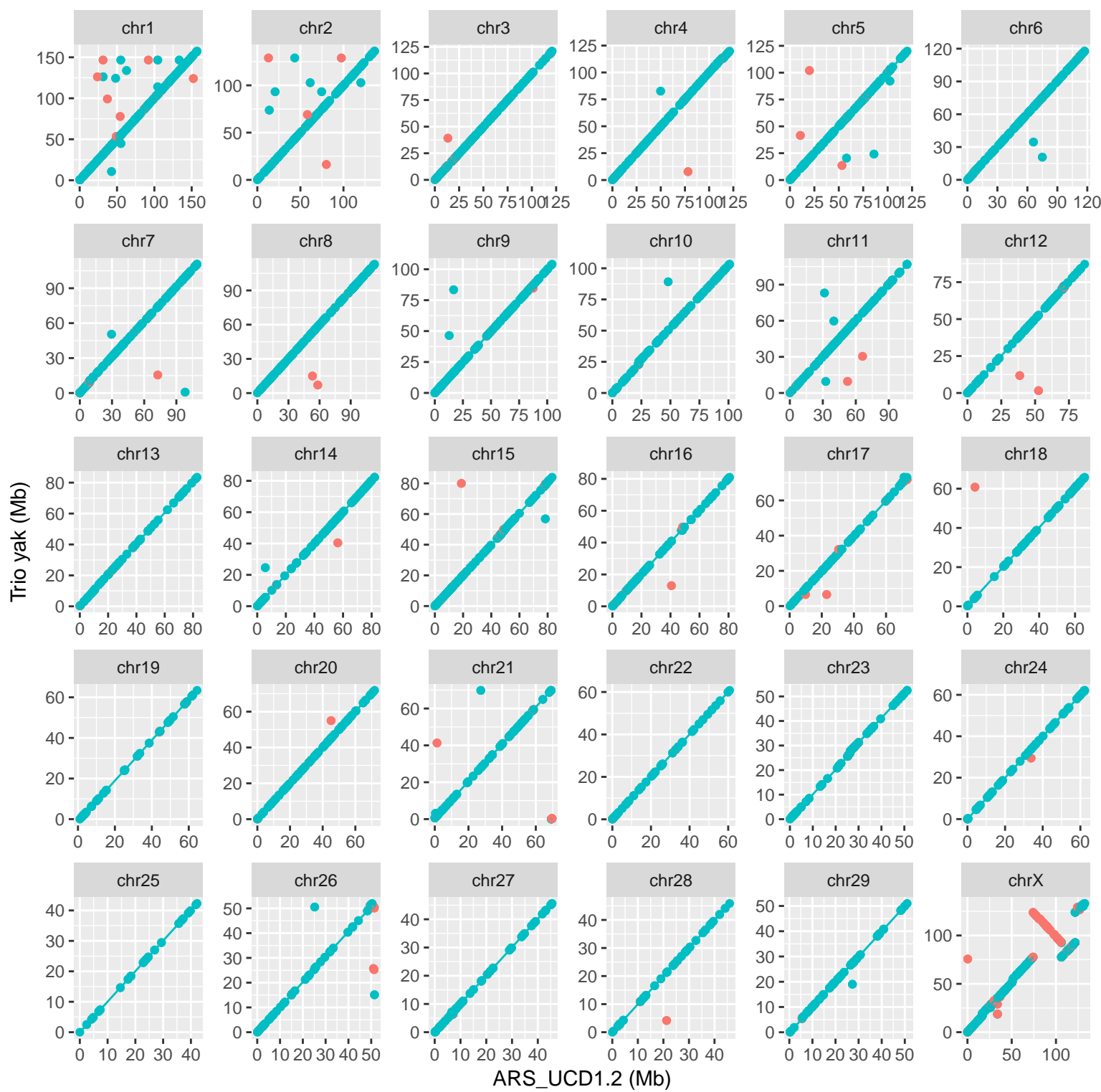

Supplement: giaa029_Supplemental_Tables_and_Figures [file giaa029_supplemental_tables_and_figures.zip › Supp_Fig_S4.pdf]

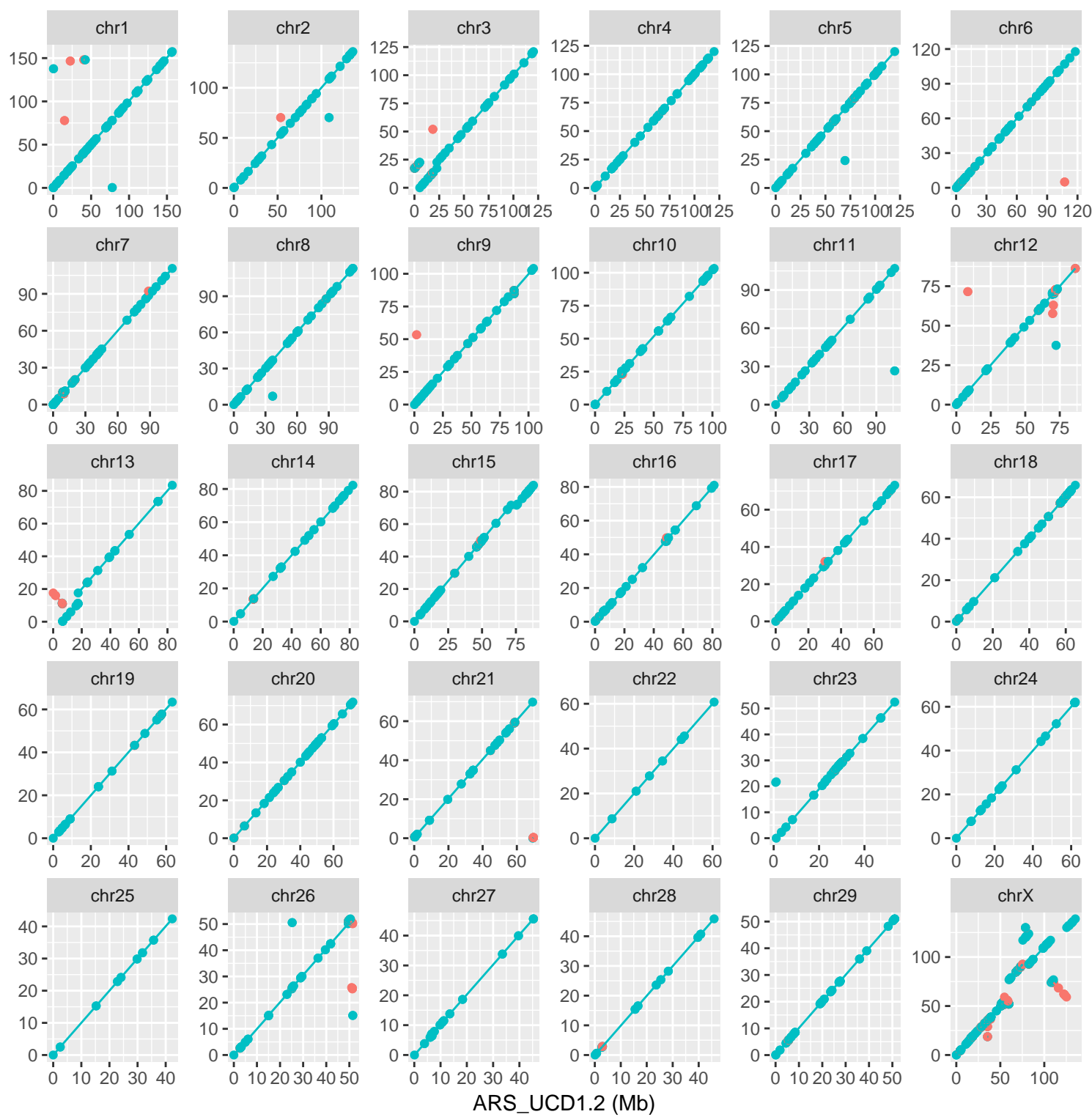

Supplement: giaa029_Supplemental_Tables_and_Figures [file giaa029_supplemental_tables_and_figures.zip › Supp_Fig_S5.pdf]
