# Supplementary material for: Continuous chromosome-scale haplotypes assembled from a single interspecies F1 hybrid of yak and cattle
Source: Gigascience. 2020 Apr 3;9(4):giaa029. doi: 10.1093/gigascience/giaa029 (PMC7118895; doi:10.1093/gigascience/giaa029)
Supplement: giaa029_Response_to_Reviewer_Comments_Original_Submission [file giaa029_response_to_reviewer_comments_original_submission.pdf]

Author's Response To Reviewer Comments

Close

Reviewer #1: In this manuscript, Rice et al applied the recently developed trio binning technology to an interspecies F1 hybrid of yak (Bos grunniens) and cattle (Bos taurus) and generated high-quality genome assemblies for both parental species simultaneously. Specifically, they sequenced both parents with 30-40X Illumina short reads and their offspring with 125X PacBio long reads. They first used the Illumina short reads from the two parents to identify 21-mers unique to each parent, then they used these unique 21-mers to sort nearly all PacBio long reads into maternal or paternal bins before assembly, so that the assembly process is greatly simplified. Although the trio binning technology is not original in this study, the authors maximized the performance of this technology by applying it to a cross-species hybrid with high heterozygosity. As a result, the authors achieved two haplotype-resolved reference genomes (one for yak and the other for cattle) with impressive continuity.

It is really impressive to see genome assemblies with contig N50 > 70 Mb, and so many chromosome arms are comprised of a single contig. Undoubtedly, the haploid genome assemblies of yak and cattle generated in this study represent the most continuous animal assemblies reported so far. This study also presents a practical example for generating high-quality assemblies for any pair of species that can interbreed to produce viable offspring. In general, the manuscript is well organized and easy to follow. I recommend the publication of this manuscript after some minor comments as listed below are addressed.

We thank the reviewer for the thorough and encouraging review of our manuscript. We appreciate all of the comments and suggested edits, which we find greatly improve the clarity of the manuscript. We address each individual point below.

Page 4, paragraph 3, "15 in maternal and 12 in paternal out of 29": it would be appreciated if the authors could indicate directly which 15 maternal and 12 paternal chromosomes are comprised of a single contig in Fig. 1g and 1h.

We thank the reviewer for this suggestion, which we find makes this figure much easier to interpret as the chromosome ideograms are small. We have noted these chromosomes with asterisks and updated the figure caption.

Table S3: Please explain what "Repeat Consistent", "Repeat Complex" and "No Repeat" represent in this table.

We agree that this table requires a better caption. We have added footnotes to explain these three terms.

Table S4: It is a bit ambiguous what the counts in this table mean. Given that there are 402 gaps identified on the ARS-UCD1.2 reference assembly, there should be 804 gap-flanking regions subject to the intersection of repetitive elements, right? So, do they mean the number of repeat loci (e.g. LINE/L1) found in all the 804 gap-flanking regions, or do they mean the number of gap-flanking regions containing this class of repeat?

We have added the following caption to the table:

Values are the number of repetitive elements identified in gap flanking regions or the intervening sequence between flanking sequences.

Page 7, paragraph 2, last sentence: It is a bit hard to understand how the data in Table S5 support the finding of "Inconsistency of flanking elements around gaps in the sire and dam assemblies" in the main text. Table S5 shows the number of ARS-UCD1.2 gaps which are consistently (or not consistently) closed in the yak and cattle assemblies, but it seems to show nothing about flanking elements around gaps.

To help clarify this sentence we have changed it to read as follows:

Inconsistency of the closure status of gaps in the sire and dam assemblies (Supplementary Table S5) suggests that some of these regions may have been sites of non-allelic homologous recombination that had occurred after the divergence between Bos taurus and Bos grunniens.

Page 8, the last paragraph of results: The authors claim that "The trio assemblies of the cattle and yak haplotype both contain all four subclasses of BOLA in a single contig." This is undoubtedly a good indicator of a high-quality assembly. However, there are no data or figure supporting this result in the manuscript. This is also the case for the coat-color gene KIT.

We thank the reviewer for pointing out the absence of supporting evidence for these claims. We have added a supplementary figure (S6) to the manuscript illustrating the BOLA locus compared to ARS_UCD1.2. However, KIT is also on a single contig in ARS_UCD1.2, so we have decided that pointing out the contiguity of KIT in our assemblies is not useful as it is the same as rather than an improvement over existing work. We have removed discussion of KIT from the manuscript.

According to the Methods section, the authors also generated some RNA-seq data in this study. But what species and tissues were subject to RNA-seq are not clearly indicated. It is also unclear what analyses have been done with these RNA-seq data.

We thank the reviewer for pointing out our oversight in the discussion of these data. We collected RNA-seq data to deposit in SRA to improve NCBI's gene annotation of the reference genome, and included these methods in our manuscript with the idea that all datasets associated with this manuscript and uploaded to public repositories should be described in this manuscript. However, after further discussion and based on the fact that both reviewers were bothered by the lack of any discussion of RNA-seq in the Results, we have decided that other readers are also likely to find this confusing and these methods are best left out of the manuscript.

Reviewer #2: In this study, Rice et al. applied trio binning to produce the most continuous haplotype-resolved assemblies for an interspecies F1 hybrid of yak (Bos grunniens) and cattle (Bos taurus). This manuscript assemblies an F1 hybrid from two divergent parental genomes by their own trio binning methods, and significantly improves the sequence quality. Although this work shows many advantages of trio binning method and higher-quality sequences, it just focuses on sequence assembly, and has numerous similar results or repetitive findings as compared to the paper by Koren et al., 2018. The biggest issue is that we did not see something new in this work. Furthermore, several points were confusing and needed to be addressed.

We thank the reviewer for the critical but helpful review of our manuscript. While we concede that the manuscript has overlap with Koren et al. in terms of methods, we contend that the application of this method to an interspecies hybrid, and the results of this application, constitute a significant advancement that merits publication for the following reasons: (a) the increased heterozygosity present in an interspecies hybrid allows better distinction between haplotypes, leading to a level of continuity not currently possible with any other method for diploid assembly of large genomes; (b) while intraspecies trio binning produces two reference genomes for a single species, interspecies trio binning produces one reference genome each for two different species from a single individual, of which there are no other examples in the literature to our knowledge; and (c) although a high-quality reference genome of cattle existed prior to our work, the new yak reference we present is several orders of magnitude more complete, contiguous, and continuous than the one published in Qiu et al. 2012. Nonetheless, we appreciate the critique as it has helped us consider how we can better explain the novelty of this work in our manuscript. We have edited our Conclusion accordingly.

Responses to individual points are below:

1. Which tissues are used for RNA-seq and how many libraries and data were sequenced? Why the authors performed RNA-seq? In the results section, we can't find any information about RNA-seq.

As noted in response to Reviewer 1,we collected RNA-seq data to deposit in SRA to improve NCBI's gene annotation of the reference genome. These methods were included in our manuscript with the idea that all datasets associated with this manuscript and uploaded to public repositories should be described in this manuscript. However, after further discussion and based on the fact that both reviewers were bothered by the lack of any discussion of RNA-seq in the Results, we have decided that other readers

are also likely to find this confusing and these methods are best left out of the manuscript.

2. How did the authors get the telomeric repeat location of the genome?

We thank the reviewer for noticing the absence of an explanation of this in our Methods. We have added a Methods subsection describing our technique and added the script we used to github (https://github.com/esrice/misc-tools/blob/master/count_telo_repeats.py).

3. The authors using short reads of twelve yaks and cattle to prove the two assemblies were not likely to be haplotype switch errors. Only SNP rate was selected as the criterion of judgment. More evidences should be provided.

We agree that ideally, a phasing analysis would use additional metrics beyond SNP rate to measure the evolutionary distance between two individuals in a given window of the genome. However, many of the datasets we used for this analysis are too low-coverage (~10x) to confidently call anything besides SNPs, and we are not aware of high-coverage sequencing data for a panel of this size containing both yaks and cattle from verified breeds. Further, we do not believe that analysis of more complex variants is necessary. The SNPs clearly demonstrated regions of introgression and further, SNPs are commonly used to deduce ancestry of segments of genomes in the literature (see, for example, our citation Medugorac et al. 2017).

4. More gaps were filled in the paternal assembly than the maternal assembly using the PBJelly pipeline, but the contig N50s of the maternal assembly improved much more than the paternal assembly. Why?

We greatly appreciate the reviewer's attention to detail in noticing the seeming discrepancy between the changes in number of gaps versus in contig N50. We were initially also intrigued by this fact after reading this review, but after further consideration, we believe that it is most likely an artefact of the uneven distribution of gaps throughout the assemblies and the discrete nature of N50. For example, filling ten gaps between pairs of 1Mb contigs, while certainly an improvement, will not have any effect on contig N50 if it was already >2Mb before filling these gaps, whereas filling a single gap between two large contigs about the size of the pre-gap-filling contig N50 could double the contig N50.

5. The locus and integrity of BOLA and KIT sequences should be exhibited as Figures (e.g. IGV or UCSC) for better understanding these results.

We thank the reviewer for pointing out the absence of supporting evidence for these claims. We have added a supplementary figure (S6) to the manuscript illustrating the BOLA locus compared to ARS_UCD1.2. However, KIT is also on a single contig in ARS_UCD1.2, so we have decided that pointing out the contiguity of KIT in our assemblies is not useful as it is the same as rather than an improvement over existing work. We have removed discussion of KIT from the manuscript.

6. Do you have some data from other full-sibling offspring? Assembling and comparing these samples to estimate recombination and exchange rates in each generation might be reasonable.

Esperanza was privately bred, and to our knowledge, no full siblings exist. We agree that sequencing one or more siblings of an individual with a fully phased genome assembly would be an excellent way to study the genomic landscape of meiotic recombination; however, it is not be feasible to breed siblings at this time. Moreover, obtaining a statistically useful estimate of recombination and exchange rates in the manner suggested would require a substantial number of full-sib offspring, which is not practical in a large animal species with a 5-6 year generation interval and low success rate of interspecies breeding.

7. What about assessing molecular mechanisms behind heterosis using trio binning method? Since you can distinguish paternal or maternal origin for all haplotypes of offspring, it might be useful to predict loci leading to heterosis in animals or plants.

We agree that the ability to create phased genome assemblies of interspecies hybrid diploid organisms presents an excellent opportunity to explore the molecular mechanisms of various aspects of sexual reproduction such as meiotic recombination as well as to aid in the understanding of allele-specific expression, imprinting, and epistasis. However, work to better understand the mechanisms of heterosis will require the collection of a significant amount of data (genotype and phenotype) from a variety of related admixed and un-admixed individuals. This work would be beyond the scope of the present manuscript, which is focused on describing the results of applying the trio binning method to an

interspecies cross for the first time, to generate extremely high-quality assemblies of both species. We thank the reviewer for the thoughtful suggestion just the same.

Close
