# Supplementary material for: Continuous chromosome-scale haplotypes assembled from a single interspecies F1 hybrid of yak and cattle
Source: Gigascience. 2020 Apr 3;9(4):giaa029. doi: 10.1093/gigascience/giaa029 (PMC7118895; doi:10.1093/gigascience/giaa029)
Supplement: giaa029_Response_to_Reviewer_Comments_Revision_1 [file giaa029_response_to_reviewer_comments_revision_1.pdf]

Author's Response To Reviewer Comments

Close

We have made all requested revisions.

Close
