# Supplementary material for: Continuous chromosome-scale haplotypes assembled from a single interspecies F1 hybrid of yak and cattle
Source: Gigascience. 2020 Apr 3;9(4):giaa029. doi: 10.1093/gigascience/giaa029 (PMC7118895; doi:10.1093/gigascience/giaa029)
Supplement: giaa029_Reviewer_1_Report_Original_Submission — Qiye Li -- 11/15/2019 Reviewed [file giaa029_reviewer_1_report_original_submission.pdf]

Reviewer Report

Title: Continuous chromosome-scale haplotypes assembled from a single interspecies F1 hybrid of yak and cattle

Version: Original Submission **Date:** 11/15/2019

Reviewer name: Qiye Li

Reviewer Comments to Author:

In this manuscript, Rice et al applied the recently developed trio binning technology to an interspecies F1 hybrid of yak (Bos grunniens) and cattle (Bos taurus) and generated high-quality genome assemblies for both parental species simultaneously. Specifically, they sequenced both parents with 30-40X Illumina short reads and their offspring with 125X PacBio long reads. They first used the Illumina short reads from the two parents to identify 21-mers unique to each parent, then they used these unique 21-mers to sort nearly all PacBio long reads into maternal or paternal bins before assembly, so that the assembly process is greatly simplified. Although the trio binning technology is not original in this study, the authors maximized the performance of this technology by applying it to a cross-species hybrid with high heterozygosity. As a result, the authors achieved two haplotype-resolved reference genomes (one for yak and the other for cattle) with impressive continuity.
It is really impressive to see genome assemblies with contig N50 > 70 Mb, and so many chromosome arms are comprised of a single contig. Undoubtedly, the haploid genome assemblies of yak and cattle generated in this study represent the most continuous animal assemblies reported so far. This study also presents a practical example for generating high-quality assemblies for any pair of species that can interbreed to produce viable offspring. In general, the manuscript is well organized and easy to follow. I recommend the publication of this manuscript after some minor comments as listed below are addressed.
Page 4, paragraph 3, "15 in maternal and 12 in paternal out of 29": it would be appreciated if the authors could indicate directly which 15 maternal and 12 paternal chromosomes are comprised of a single contig in Fig. 1g and 1h.
Table S3: Please explain what "Repeat Consistent", "Repeat Complex" and "No Repeat" represent in this table.
Table S4: It is a bit ambiguous what the counts in this table mean. Given that there are 402 gaps identified on the ARS-UCD1.2 reference assembly, there should be 804 gap-flanking regions subject to the intersection of repetitive elements, right? So, do they mean the number of repeat loci (e.g. LINE/L1) found in all the 804 gap-flanking regions, or do they mean the number of gap-flanking regions containing this class of repeat?
Page 7, paragraph 2, last sentence: It is a bit hard to understand how the data in Table S5 support the finding of "Inconsistency of flanking elements around gaps in the sire and dam assemblies" in the main text. Table S5 shows the number of ARS-UCD1.2 gaps which are consistently (or not consistently) closed in the yak and cattle assemblies, but it seems to show nothing about flanking elements around gaps.
Page 8, the last paragraph of results: The authors claim that "The trio assemblies of the cattle and yak

haplotype both contain all four subclasses of BOLA in a single contig." This is undoubtedly a good indicator of a high-quality assembly. However, there are no data or figure supporting this result in the manuscript. This is also the case for the coat-color gene KIT.
According to the Methods section, the authors also generated some RNA-seq data in this study. But what species and tissues were subject to RNA-seq are not clearly indicated. It is also unclear what analyses have been done with these RNA-seq data.

Level of Interest

Please indicate how interesting you found the manuscript: Choose an item.

Quality of Written English

Please indicate the quality of language in the manuscript: Choose an item.

Declaration of Competing Interests

Please complete a declaration of competing interests, considering the following questions:

- Have you in the past five years received reimbursements, fees, funding, or salary from an organisation that may in any way gain or lose financially from the publication of this manuscript, either now or in the future?
- Do you hold any stocks or shares in an organisation that may in any way gain or lose financially from the publication of this manuscript, either now or in the future?
- Do you hold or are you currently applying for any patents relating to the content of the manuscript?
- Have you received reimbursements, fees, funding, or salary from an organization that holds or has applied for patents relating to the content of the manuscript?
- Do you have any other financial competing interests?
- Do you have any non-financial competing interests in relation to this paper?

If you can answer no to all of the above, write 'I declare that I have no competing interests' below. If your reply is yes to any, please give details below.

I declare that I have no competing interests

I agree to the open peer review policy of the journal. I understand that my name will be included on my report to the authors and, if the manuscript is accepted for publication, my named report including any attachments I upload will be posted on the website along with the authors' responses. I agree for my report to be made available under an Open Access Creative Commons CC-BY license (http://creativecommons.org/licenses/by/4.0/). I understand that any comments which I do not wish to

be included in my named report can be included as confidential comments to the editors, which will not be published.

Choose an item.

To further support our reviewers, we have joined with Publons, where you can gain additional credit to further highlight your hard work (see: https://publons.com/journal/530/gigascience). On publication of this paper, your review will be automatically added to Publons, you can then choose whether or not to claim your Publons credit. I understand this statement.

Yes Choose an item.
