# Supplementary material for: Continuous chromosome-scale haplotypes assembled from a single interspecies F1 hybrid of yak and cattle
Source: Gigascience. 2020 Apr 3;9(4):giaa029. doi: 10.1093/gigascience/giaa029 (PMC7118895; doi:10.1093/gigascience/giaa029)
Supplement: giaa029_Reviewer_2_Report_Original_Submission — Zhonglin Tang, Ph.D. -- 11/17/2019 Reviewed [file giaa029_reviewer_2_report_original_submission.pdf]

Reviewer Report

Title: Continuous chromosome-scale haplotypes assembled from a single interspecies F1 hybrid of yak and cattle

Version: Original Submission **Date:** 11/17/2019

Reviewer name: Zhonglin Tang, Ph.D.

Reviewer Comments to Author:

In this study, Rice et al. applied trio binning to produce the most continuous haplotype-resolved assemblies for aninterspecies F1 hybrid of yak (Bos grunniens) and cattle (Bos taurus). This manuscript assemblies an F1 hybrid from two divergent parental genomes by their own trio binning methods, and significantly improves the sequence quality. Although this work shows many advantages of trio binning method and higher-quality sequences, it just focuses on sequence assembly, and has numerous similar results or repetitive findings as compared to the paper by Koren et al., 2018. The biggest issue is that we did not see something new in this work. Furthermore, several points were confusing and needed to be addressed.

1. Which tissues are used for RNA-seq and how many libraries and data were sequenced? Why the authors performed RNA-seq? In the results section, we can't find any information about RNA-seq.
2. How did the authors get the telomeric repeat location of the genome?
3. The authors using short reads of twelve yaks and cattleto prove the two assemblies were not likely to be haplotype switch errors. Only SNP rate was selected as the criterion of judgment. More evidences should be provided.
4. More gaps were filled in the paternal assembly than the maternal assembly using the PBJelly pipeline, but the contig N50s of the maternal assembly improved much more than the paternal assembly. Why?
5. The locus and integrity of BOLA and KIT sequences should be exhibited as Figures (e.g. IGV or UCSC) for better understanding these results.
6. Do you have some data from other full-sibling offspring? Assembling and comparing these samples to estimate recombination and exchange rates in each generation might be reasonable.
7. What about assessing molecular mechanisms behind heterosis using trio binning method? Since you can distinguish paternal or maternal origin for all haplotypes of offspring, it might be useful to predict loci leading to heterosis in animals or plants.

Level of Interest

Please indicate how interesting you found the manuscript: Choose an item.

Quality of Written English

Please indicate the quality of language in the manuscript: Choose an item.

Declaration of Competing Interests

Please complete a declaration of competing interests, considering the following questions:

- Have you in the past five years received reimbursements, fees, funding, or salary from an organisation that may in any way gain or lose financially from the publication of this manuscript, either now or in the future?
- Do you hold any stocks or shares in an organisation that may in any way gain or lose financially from the publication of this manuscript, either now or in the future?
- Do you hold or are you currently applying for any patents relating to the content of the manuscript?
- Have you received reimbursements, fees, funding, or salary from an organization that holds or has applied for patents relating to the content of the manuscript?
- Do you have any other financial competing interests?
- Do you have any non-financial competing interests in relation to this paper?

If you can answer no to all of the above, write 'I declare that I have no competing interests' below. If your reply is yes to any, please give details below.

No, I declare that I have no competing interests.

I agree to the open peer review policy of the journal. I understand that my name will be included on my report to the authors and, if the manuscript is accepted for publication, my named report including any attachments I upload will be posted on the website along with the authors' responses. I agree for my report to be made available under an Open Access Creative Commons CC-BY license (http://creativecommons.org/licenses/by/4.0/). I understand that any comments which I do not wish to be included in my named report can be included as confidential comments to the editors, which will not be published.

Choose an item.

To further support our reviewers, we have joined with Publons, where you can gain additional credit to further highlight your hard work (see: https://publons.com/journal/530/gigascience). On publication of this paper, your review will be automatically added to Publons, you can then choose whether or not to claim your Publons credit. I understand this statement.

Yes Choose an item.
